# Thermodynamics at Solid–Liquid Interfaces

**DOI:** 10.3390/e20050362

**Published:** 2018-05-12

**Authors:** Michael Frank, Dimitris Drikakis

**Affiliations:** Department of Mechanical Aerospace and Engineering, University of Strathclyde, 75 Montrose, Glasgow G11UX, UK

**Keywords:** nanofluidics, thermal conductivity, confinement, phonons, Green–Kubo

## Abstract

The variation of the liquid properties in the vicinity of a solid surface complicates the description of heat transfer along solid–liquid interfaces. Using Molecular Dynamics simulations, this investigation aims to understand how the material properties, particularly the strength of the solid–liquid interaction, affect the thermal conductivity of the liquid at the interface. The molecular model consists of liquid argon confined by two parallel, smooth, solid walls, separated by a distance of 6.58 *σ*. We find that the component of the thermal conductivity parallel to the surface increases with the affinity of the solid and liquid.

## 1. Introduction

The properties of a thermodynamic system are the result of an amalgamation of molecular interactions. Given a large number of molecules, the microscopic behaviour averages out into seemingly constant material properties, such as thermal conductivity, heat capacity, and viscosity. Most engineering applications deal with systems that are large enough to permit a simplified, averaged perspective of the microscopic structures and processes. Statistical mechanics, the branch of physics that bridges the microscopic world of atoms to the continuum world of material properties, has been a topic of interest primarily to theoretical physicists. Over the last two decades, nanotechnologies and nanomaterials have been applied commercially, and are gradually becoming a prominent branch of engineering. The physical scales associated with nanotechologies prohibit the continuum mechanics approach.

In gases, where molecular interactions are weak, heat is primarily transferred through molecular diffusion, and interactions are modelled by perturbation theory. In electrically conducting solids, such as metals, heat transfer is usually dominated by the transfer of delocalised electrons that are free to move across the atoms of the solid. The strong bonds between solid atoms also allow heat to be transferred through collective vibrations, which are quantum mechanically described by quasiparticles called phonons. The distinct behaviour of gases and solids has been studied extensively using analytical models for the thermodynamics of such systems.

Liquids exhibit more a complex behaviour. The fluidity of liquids is reminiscent of gases, but their density more closely resembles that of solids. While liquids undergo molecular diffusion, the strong molecular interactions enable the transfer of heat through phonons. Of course, the general lack of symmetry and constant deformation of liquids results in a large number of scattering events that give liquids the diffusive and isotropic nature of their heat transfer.

Frenkel [1] was the first to point out that the distinction between solids and liquids is based on loose terminology. While his ideas were perceived as too extreme at the time, a body of experimental work has since validated his theories [2,3,4,5]. Recent studies have attempted to establish a phonon-based theory for liquid thermodynamics [6,7,8] through the derivation of analytical models for the energy and heat capacity of liquids; their predictions are in general agreement with experiments.

Understanding the microscopic behaviour of liquids becomes more important in nanotechnologies, where interfacial effects dominate the system. Solid–liquid interactions introduce liquid stratification close to the solid surface, altering properties such as viscosity [9,10,11], diffusivity [12], and thermal conductivity [12]. Stratification can also change the vibrational properties of the liquid, which can impact the thermal conductance across an interface [13,14,15,16].

In a previous publication [17], we have shown that the component of the thermal conductivity of a confined liquid parallel to a smooth surface is significantly greater than the thermal conductivity of its bulk equivalent. We attributed this increase to the greater phonon mean free path, a result of the greater structural order and larger relaxation time of liquids in the vicinity of the wall.

In this paper, we investigate how the the strength of the solid–liquid interactions affects the propagation of phonons, and thermal conductivity within a nanochannel. A more comprehensive analysis is presented, and the implications, as well as future considerations of the solid-like behaviour of liquids is discussed.

## 2. Theory of Phonons

Atoms in a solid are bounded by each other through an energy potential. A frequently used analogy is that of masses connected by springs. Perturbing an atom leads to waves travelling at the speed of sound, transferring heat across the crystal lattice. Assuming relatively small displacements, we can decompose the system into a number of harmonic oscillators. These vibrational modes will have different frequencies, wavenumbers, and polarizations. Waves oscillating perpendicular to the direction of propagation are called transverse waves, whereas waves oscillating in the same direction as that of propagation are called longitudinal waves.

The energy of a classical oscillator is a continuous function of the amplitude of oscillation. However, quantum mechanically, systems bounded by a potential can only assume discrete values of energy. The energy of a quantum harmonic oscillator is given by:

(1) $$E\left(\omega \right)=\hbar \omega \left(\frac{1}{2}+n\right),\;n=1,2,3...,$$

where ω is the angular frequency; and *n* is the quantum number of the oscillator. The discretized nature of the energy permits a different interpretation: we can view the energy of a vibrational mode with a ground state $\frac{1}{2}\hbar \omega$, as the contribution of *n* particles, each of which has an energy ℏω. These particles are called phonons.

Phonons are bosons, and as such are described by the Bose–Einstein function according to which the expected number of phonons occupying a single vibrational mode is given by:

(2) $$n\left(\omega \right)=\frac{1}{\exp\left(\frac{\hbar \omega }{\kappa_{B}T}\right)-1},$$

where *T* is the temperature; and $\kappa_{B}$ is the Boltzmann constant.

Substituting Equation (2) into (1) and considering all vibrational modes, the total energy of the system yields:

(3) $$E=\sum_{\omega }=\hbar \omega \left[\frac{1}{2}+\frac{1}{\exp\left(\frac{\hbar \omega }{\kappa_{B}T}-1\right)}\right].$$

The heat capacity of a system can then be calculated by

(4) $$C_{V}=\frac{\partial E}{\partial T}.$$

At “high” temperatures, the energy of a vibrational mode is approximated by $\kappa_{B}T$ and does not depend on the vibrational frequency. The total energy of the system reduces to $3N\kappa_{B}T=3RT$, where *R* is the ideal gas constant. However, for many materials, the model is accurate only for temperatures much higher than room temperature, thus rendering the approximation impractical.

More elaborate models consider the distribution of the possible energy states of the system across the available vibrational frequencies. This is formally described by the phonon density of states, g(ω), where the number of states within an infinitesimal increment of the frequency is g(ω)dω. The total energy is given by

(5) $$E=\sum_{i}g\left(\omega_{i}\right)n\left(\omega_{i}\right).$$

The harmonic approximation provides an asymptotic limit, but is, in general, non-physical. Even in atomically flawless crystals, phonons will scatter at the boundaries of the solid. The harmonic approximation does not consider scattering events and phonons are considered to propagate uninterrupted. The phonon mean free path—the average distance travelled by a phonon prior to scattering—is infinite, thus resulting in an infinite thermal conductivity.

Anharmonicities can be taken into account by including higher order terms in the Taylor series expansion of the potential. The coefficient of thermal expansion is a good indicator of the anharmonic behaviour of a system: greater coefficients correspond to a more anharmonic behaviour. The large displacements of liquid atoms result in a large number of scattering events, as indicated by the significantly greater coefficients of thermal expansion, compared to those of solids.

Recent studies [6,7,8] have discussed the extension of the phonon theory for solids to liquids. They have treated anharmonicities by considering the thermal expansion coefficients of liquids. Based on Frenkel’s theory [1], shear waves with a frequency smaller than $\frac{1}{\tau }$ were ignored, where τ is the liquid relaxation time.

## 3. Methodology

The computational model consists of liquid argon confined in a channel of nanometer characteristic dimensions. The walls of the channel are fixed perpendicular to the *y*-direction (parallel to the xz plane) with dimensions in the *x*- and *z*-directions being $L_{x}=14.14$ σ and $L_{z}=16.33$ σ, respectively, where σ denotes the molecular diameter of liquid argon (≈0.3405 nm). $L_{y}$, the distance separating the walls, is set to 6.58 σ. Periodic boundary conditions are used along the *x*- and *z*-directions, emulating the perpetual continuation of the channel. In the *y*-direction, the boundaries coincide with the walls and fixed boundary conditions are used. Each of the two walls consists of two (111)- oriented perfect Face-Center Cubic (fcc) lattice planes with density $\rho_{wall}^{*}=4$ m$\sigma^{-3}$.

We model the interactions between liquid–liquid and liquid–solid atoms using the Lennard–Jones (*LJ*) potential:

(6) $$\nu_{ij}^{LJ}\left(r_{ij}\right)=4\varepsilon \left[{\left(\sigma /r_{ij}\right)}^{12}-{\left(\sigma /r_{ij}\right)}^{6}\right],$$

where *i*, *j* are the labels for two arbitrary particles in the system; $r_{ij}$ is their interatomic distance; and ε depth of the energy well, and controls the strength of the interaction. For computational efficiency, interatomic interactions beyond a cut-off distance $r_{c}=2.2$ σ are disregarded. The *LJ* parameters for the potential between argon particles are $\varepsilon_{ll}=1.0$ $\varepsilon =1.6539\times 10^{-21}$ *J* and $\sigma_{ll}=1.0$ σ=0.34 nm. The strength of the solid–liquid interaction is a parameter of interest to this study, and varies from 0.4 ε to 1 ε. Finally, we set $\sigma_{sl}=0.75$ σ. The mass of all atoms was set equal to $m=6.69\times 10^{-26}$ kg. We inserted 1280 argon atoms in the channel, realizing a density $\rho_{l}=0.84$ $N\sigma^{-3}$, which along with the temperature $T^{*}=0.7$ $\varepsilon k_{B}^{-1}$, represents the liquid phase of argon [18,19]. The equations of motion for the particle *i* are given by

(7) $$m\ddot{r_{i}}=-\sum_{i\neq j}\nabla \nu_{ij}^{LJ}\left(r_{ij}\right).$$

The wall particles are fixed onto their initial lattice sites by spring potentials urging them to return to their equilibrium positions $r_{0}$ via a restoring force given by

(8) $$F=-K(r_{i}-r_{0}),$$

where *K* is the wall stiffness, a parameter vital to the realistic representation of the wall. Its value determines the strength of the bonds between the wall’s particles. For the current study, the value K=500 $\epsilon \sigma^{-2}$ is used [20].

In order to control the temperature of the system, each of the fcc planes of the walls is assigned a thermostat [21]. The thermostats control the temperature, which is related to the kinetic energy of the atoms through the equipartition theorem, by multiplying the velocity of each atom with $\sqrt{\frac{T_{0}}{T(t)}}$, where T(t) is the temperature of the current timestep; and $T_{0}$ is the desired temperature. Note that scaling velocities should be avoided on atoms that are directly used for the calculation of properties. Such cases should instead employ the Nose–Hover equations to advance the system within the canonical ensemble (NVT), i.e., the number of atoms *N*, volume of the system *V* and temperature *T* remain constant. In this paper, Newton’s equations of motion (Equation (7)) is used to advance the system in time, restricting the system within the microcanonical ensemble (NVE), i.e., the number of atoms *N*, volume of the system *V*, and energy *E* remain constant. Due to the choice of statistical ensemble and the fact that the wall atoms are not used in the calculations, scaling the velocities of the solid atoms is a simple and accurate approach for retaining the temperature constant. Both channel walls are set to the same temperature, $T^{*}=0.7$ $\varepsilon k_{B}^{-1}$.

The two techniques for calculating the thermal conductivity using Molecular Dynamics (MD) are the non-equilibrium method and the Green–Kubo (GK) approach. The non-equilibrium method introduces a one-dimensional temperature gradient. The resulting heat flux is then used, in conjunction with Fourier’s law, to calculate the thermal conductivity. However, in nanometer sized samples, applying a temperature difference of the order of 10 *K* can lead to non-physical results [22,23].

The GK formalism is based on the fluctuation-dissipation theorem, i.e., the dissipation patterns of the thermal fluctuations of a system in equilibrium are used to calculate the thermal conductivity. The thermal conductivity is calculated by

(9) $$\lambda =\frac{1}{V\kappa_{B}T^{2}d}\int_{0}^{\infty }\langle J(0).J(t)\rangle dt,$$

where λ is the thermal conductivity; *V* is the volume of the system; $\kappa_{B}$ is the Boltzmann constant; *d* is the number of dimensions of the system; the angled brackets indicate an autocorrelation function; and *J* is the microscopic heatflux, given by:

(10) $$J=\sum_{i=1}^{N}v_{i}E_{i}+\frac{1}{2}\sum_{i=1}^{N}\sum_{j\neq 1}^{N}r_{\mathrm{ij}}\left(F_{\mathrm{ij}}\cdot v_{i}\right)-\sum_{i=1}^{N}v_{i}h,$$

where $v_{i}$ is the velocity of particle *i*; $E_{i}$ is the total energy of particle *i*; $F_{\mathrm{ij}}$ is the interatomic force between particle *i* and *j*; and *h* is the average enthalpy of the liquid, calculated as the sum of the average kinetic energy, potential energy, and viral terms [18,24]. For a system in equilibrium, the Heat Flux Autocorrelation Function (HFACF) $\langle J(0).J(t)\rangle$ should eventually decay to zero so that its integral (and therefore the thermal conductivity) has a finite and well defined value. The main issue with this approach is that HFACF is sensitive to statistical noise, which can accumulate significantly. This is particularly true for long correlation lengths that are often required for the HFACF to converge [23,25].

The thermal conductivity of liquids in nanochannels is expected to be anisotropic. In the present model, the *x*- and *z*-directions that are parallel to the solid surface are isotropic due to the periodic boundary conditions used. The total thermal conductivity, as well as the anisotropy of the thermal conductivity in nanochannels has been discussed in other papers [12,26]. For the remainder of this paper, any reference to the HFACF and thermal conductivity imply the component in the *x*-direction. The thermal conductivity in the *z*-direction yields identical results.

**Figure 1:**
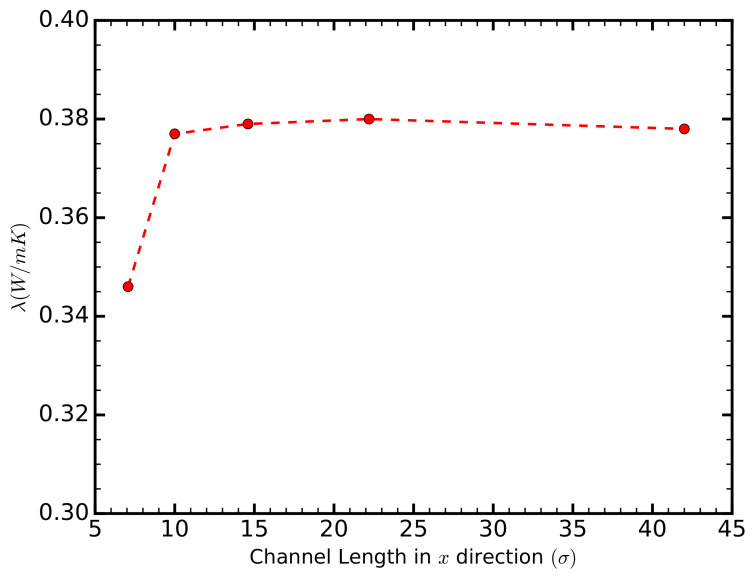
The thermal conductivity of the liquid in the *x*-direction as a function of the channel length in the same direction, $L_{x}$, for a channel of height 6.58 σ, and a strength of solid–liquid interaction $\varepsilon_{sl}=0.8$ ε. The thermal conductivity converges for channel lengths greater than 10 σ.

In this study, we calculate the thermal conductivity using the GK formalism, primarily due to the information provided by the HFACF, which sheds light on the heat transfer mechanism. The GK method has been used extensively with periodic boundary conditions [22,23,25]. However, accurate calculations require the size of the simulation box in the direction of the calculation to be comparable to the phonon mean free path McGaughey and Kaviany [23]. In order to reduce size-related uncertainties, we calculated the thermal conductivity for channels of different lengths and found it to converge for lengths greater than $L_{x}=10$ σ (Figure 1).

The characteristic time of the simulation is $\tau =\sigma \sqrt{\frac{m}{\varepsilon }}\approx 2.15$ ps. For the calculation of the thermal conductivity, the simulation step is δt=0.001τ≈2 fs. An initial equilibration phase of $2\times 10^{6}$ δt takes place to allow the temperature and energy of the system to settle. The simulations are then performed for a further $4\times 10^{7}$ δt and the positions at each timestep are taken from the microcanonical ensemble (NVE). For the calculation of the HFACF, a correlation length of $2\times 10^{5}$ δt is used giving the autocorrelation function sufficient time to decay.

The Vibrational Density of States (VDOS) are calculated as the Fourier transform of the autocorrelation function of the velocity [27]. For the calculation of the Fourier transform, we have sampled the liquid velocities every 5 δt, for a total of 4000 δt. Due to the constant restructuring of the liquid particles, the VDOS of the same system would vary slightly between different runs. To reduce such uncertainty, we run each case 10 times, starting with different atomic velocities and positions, and took the average value for each frequency.

## 4. Results

**Figure 2:**
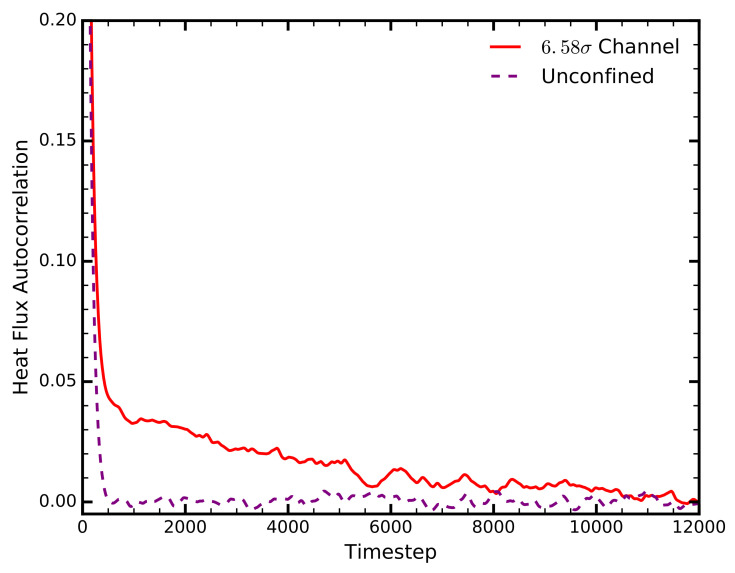
The Heat Flux Autocorrelation Function for bulk and confined liquid argon, for $\varepsilon_{sl}=0.8$ ε. Reconstructed from Frank and Drikakis [17].

The HFACF provides qualitative and quantitative information on the average behaviour of phonons in a system. We observe a different pattern of energy dissipation between the liquid in bulk, and its confined equivalent (Figure 2). The HFACF in the bulk liquid decays monotonically within a very short time frame, indicative of the diffusive nature of liquids. Under confinement, however, the HFACF follows a two-stage decay: an initial rapid stage, coinciding with that of the liquid in bulk, and a second, more gradual decay. This two-stage decay, initially observed in crystal solids, encapsulates the diffusive component of the thermal conductivity, as well as the component due to long-range phonons [23].

We have previously shown that the different vibrational properties of confined liquids is due to the ordered liquid structure imposed by the solid surfaces [17]. The thermal conductivity in the direction parallel to the channel wall increases, as a result of the larger phonon relaxation time and mean free path. However, we did not consider the effect of different materials. A first step in doing so is to understand how the nature of the solid–liquid interactions affects the thermal conductivity.

**Figure 3:**
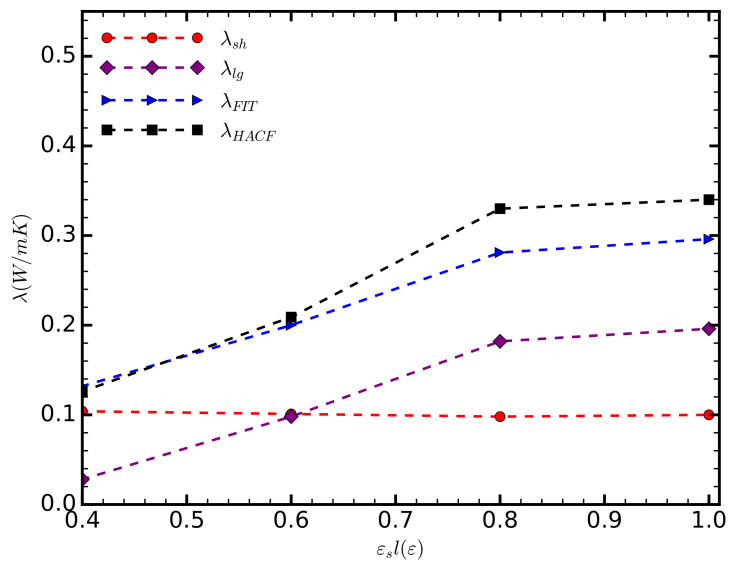
The component of the thermal conductivity in the direction parallel to the surface, as a function of the strength of the solid–liquid interaction. The different curves correspond to the thermal conductivity as calculated by directly integrating HFACF (■); the thermal conductivity as calculated by fitting a sum of two exponential functions on the HFACF (◆); and the decomposed short- (●), long-range (◆) components of the thermal conductivity.

The thermal conductivity, as calculated directly by integrating HFACF, increases along with the strength of the solid–liquid interaction (■ in Figure 3). Initially, the thermal conductivity increases linearly. As the adhesive and cohesive forces balance out, i.e., $\varepsilon_{sl}=1$ ε, we observe a reduction in the gradient of the thermal conductivity, tending to an asymptotic state; further simulations, however, are required to confirm the above. For the range of cases considered here, the thermal conductivity in the direction parallel to the channel walls increases approximately three-fold compared to the thermal conductivity of bulk argon (0.132 W/mK) [28].

To better appreciate the underlying mechanisms of heat transfer, we decompose the thermal conductivity by fitting a sum of two exponential functions onto the HFACF. This function is given by

(11) $$\begin{array}{c} \langle q\left(t\right)\cdot q\left(0\right)\rangle =A_{sh}\exp\left(-t/\tau_{sh}\right)+A_{lg}\exp\left(-t/\tau_{lg}\right), \end{array}$$

where the indices ${}_{s}h$ and ${}_{l}g$ stand for short-range and long-range, respectively; *A* and τ are the average strength and relaxation times of phonons in the system. The short-range, $\lambda_{sh}$, and long-range, $\lambda_{lg}$, components of the thermal conductivity can then be calculated by

(12) $$\lambda_{FIT}=\left(A_{sh}\tau_{sh}+A_{lg}\tau_{lg}\right)=\lambda_{sh}+\lambda_{lg}.$$

As the strength of the solid–liquid interaction increases, the short-range component of thermal conductivity decreases, albeit very slightly (● in Figure 3). We attribute this decrease to the adhesive forces immobilizing the liquid atoms, thus reducing molecular diffusion and the associated heat transfer. However, this change is very small and the short range component of the thermal conductivity remains practically equal to that of the unconfined liquid argon.

The correlation between the total thermal conductivity and the strength of the solid–liquid interaction is predominantly due to the contribution of long-range phonons (Figure 3). For $\varepsilon_{sl}=0.4$ ε, the lowest value considered here, $\lambda_{lg}$ almost vanishes. As $\varepsilon_{sl}$ increases, $\lambda_{lg}$ follows a trend similar to that of the total thermal conductivity. For values greater than $\varepsilon_{sl}=0.6$ ε, the long-range phonons become the dominant mechanism of heat transfer parallel to the channel.

Note the discrepancy (≈6%) between $\lambda_{HFACF}$ and $\lambda_{FIT}$, for $\varepsilon_{sl}=0.8$ ε and $\varepsilon_{sl}=1.0$ ε. We assume that the fitted values are more precise, as the HFACF is known to accumulate error, particularly when long correlation lengths are used [29]. In fact, fitting has previously been used as a means to filter unwanted noise [25].

**Figure 4:**
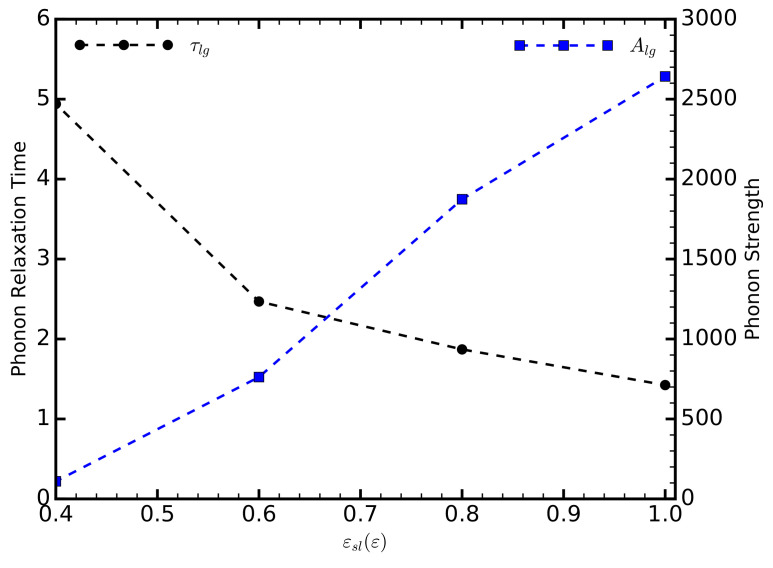
The relaxation time, and strength of the long-range phonons, as a function of the strength of the solid–liquid interaction.

**Figure 5:**
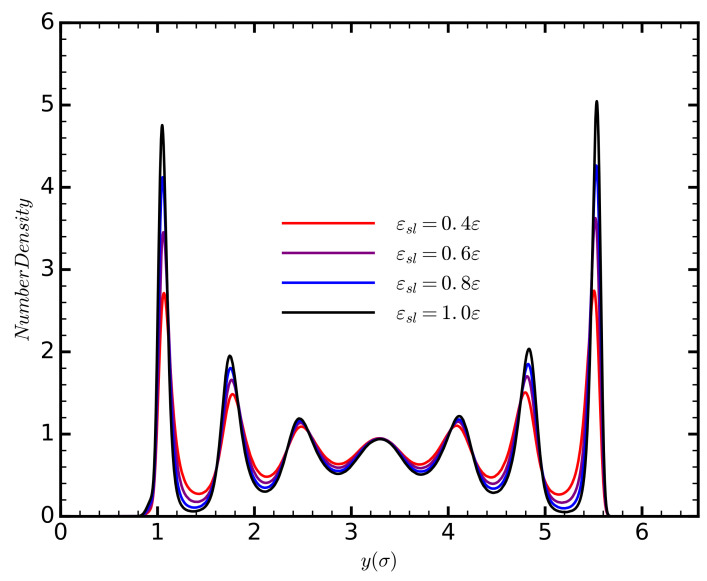
The density profiles of the liquid for different values of the strength of the solid–liquid interaction.

The thermal conductivity is influenced by the number of phonons available in the system, the frequencies of the phonons, and the phonon mean free path. To delineate these effects in our system, and how they contribute to the thermal conductivity, we start by considering the phonon relaxation time, τ, and strength, *A*, obtained from the fitting Equation (11) on the HFACF data (Figure 4). The phonon relaxation time decreases as the strength of the solid–liquid interaction increases. This effect is either due to an increase in the phonon velocity causing the phonon to cover the distance of the mean free path faster, or to an increased occurrence of scattering events, which would reduce the phonon mean free path. Increasing the strength of the solid–liquid interaction increases the density of the liquid layers (Figure 5), thus decreasing the interatomic spacing of the liquid atoms. This would decrease the phonon group velocity given by $v_{g}=\alpha \sqrt{\frac{C}{m}}$, where α is the interatomic spacing; and *C* is the spring potential between the atoms, defined as the second derivative of the potential energy with respect to the atomic displacement. Contrary to our observations, a decreasing phonon velocity would result in an increasing relaxation time. It is more likely that the reduction of $\tau_{lg}$ is due to an increased occurrence of scattering events. This claim, however, requires further investigation, as the increasing density of the liquid could also influence the value of *C*.

While the relaxation time decreases, the phonon strength increases. For the values of $\varepsilon_{sl}$ considered here, *A* increases in a linear fashion. This suggests a change in the vibrational properties of the liquids.

**Figure 6:**
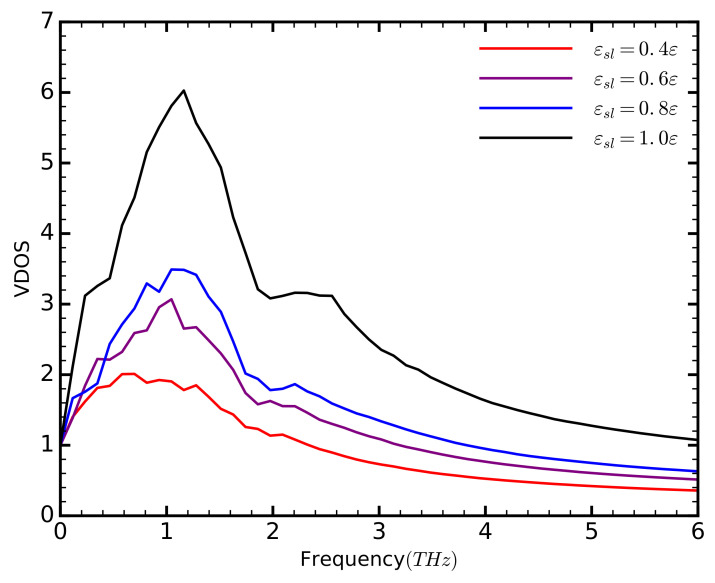
The vibrational density of states for different values of $\varepsilon_{sl}$.

The increase in phonon strength is due to a larger number of available vibrational states (Figure 6). The VDOS increase with increasing $\varepsilon_{sl}$, although we do not observe any significant differences in the frequencies of the energy peaks. Thus, we attribute the increase of the thermal conductivity to a larger number of phonon modes available in the system. The increase of the VDOS, however, does not match the trend of the thermal conductivity. Although the VDOS exhibits the largest increase between the cases with $\varepsilon_{sl}=0.8$ ε and $\varepsilon_{sl}=1$ ε , the thermal conductivity exhibits only a slight increase. We attribute this to an increase in Umklapp scattering, which generally increases with the number of phonons. This is similar to the effects of temperature on the thermal conductivity of Carbon Nanotubes (CNTs) [30,31]: as the temperature increases beyond absolute zero, more phonon modes are probed, which in turn increase the thermal conductivity. As the number of phonons increases, so does the Umklapp scattering. Beyond a temperature value, usually close to room temperature, the destructive effects of scattering overcome the constructive effects of the additional phonon modes, and the thermal conductivity starts to decrease. We expect a similar behavior here. As the affinity of the solid and liquid continues to increases, the thermal conductivity will eventually plateau or even decrease.

This study highlights a number of directions for further pursuit. For example, the effects of temperature of the system on the thermal conductivity of the liquid require further elucidation. On the one hand, the additional energy corresponding to a higher temperature could excite additional phonon modes, thus increasing the thermal conductivity. On the other hand, an increasing temperature could have a negative effect on the thermal conductivity by increasing Umklapp scattering, or by breaking the structure of the liquid layers next to the solid walls.

Other solid properties, such as the density and crystallographic direction of the solid surface, can also affect the density profiles of the liquid in a fashion similar to the strength of the solid–liquid interaction [32]. Would a similar change in the density profiles, resulting by changing these different properties, produce a similar change in thermal conductivity?

Although the viscoelastic nature of confined liquids in smooth geometries has been demonstrated experimentally, will this behavior persist in the presence of surface roughness? Recent studies have shown that wall irregularities break down the liquid layers close to the solid [33], altering the thermodynamic properties of the liquid such as viscosity at the interface [11].

Finally, how does the behavior of phonons in confined liquids affect other thermodynamic properties or flow dynamics, which have already been shown to differ in nanochannels [12]? Can this theory fit in the broader theme of micro- and nano-fluidics and build upon existing mathematical frameworks [34] on the behavior of such systems?

## 5. Conclusions

Using MD simulations, we have investigated the effect of the strength of the solid–liquid interaction on the component of the thermal conductivity of a confined liquid parallel to the channel walls. The main findings of the paper are summarized below:

1. The thermal conductivity increases with increasing the strength of the solid–liquid interaction. Up to $\varepsilon_{sl}=0.8$ ε, this increase is linear. The slope of the thermal conductivity between $\varepsilon_{sl}=0.8$ ε and $\varepsilon_{sl}=1$ ε decreases, suggesting a possible asymptotic state. However, further simulations covering a broader range of solid–liquid interactions are required to confirm the above.
2. We attribute the observed increase of the thermal conductivity to a larger number of phonon states available in the system. The thermal conductivity does not, however, follow the trend of the VDOS. We attribute this to the Umklapp scattering, which is known to increase as the number of phonons increases. Scattering events have a negative effect on thermal conductivity; this is probably the reason for the decreasing slope of the thermal conductivity for $\varepsilon_{sl}>0.8$ ε.
3. We speculate that as the strength of the solid–liquid interaction further increases and more phonon modes become available, an increase in scattering will cause the thermal conductivity to reach an asymptotic state and even decrease. A similar phenomenon is observed with respect to the temperature effects on the thermal conductivity of CNTs.

Subject to further investigation, we believe that a deeper level of understanding of the thermodynamics and heat transfer at solid–liquid interfaces can have an impact in engineering and industry. A direct application would be for the design of more efficient nano-channel heat sinks. As thermal management is currently a bottleneck for adding or improving the functionality of high demanding applications, such as avionics, better cooling techniques could have a significant impact on these industries. Extending our findings to the broader theme of nanofluidics could also provide tools for optimizing nanotechnologies, which would benefit a wide range of different industries.
